# Point-of-Care Viral Load Testing to Manage HIV Viremia During the Rollout of Dolutegravir-Based ART in South Africa: A Randomized Feasibility Study (POwER)

**DOI:** 10.1097/QAI.0000000000003212

**Published:** 2023-04-25

**Authors:** Jienchi Dorward, Yukteshwar Sookrajh, Richard Lessells, Elliot Bulo, Jessica Naidoo, Keshani Naidoo, Nicola Bodley, Mlungisi Khanyile, Claudia Jansen Van Vuuren, Pravikrishnen Moodley, Natasha Samsunder, Lara Lewis, Paul K. Drain, Gail Hayward, Christopher C. Butler, Nigel Garrett

**Affiliations:** a Nuffield Department of Primary Care Health Sciences, University of Oxford, Oxford, United Kingdom;; b Centre for the AIDS Programme of Research in South Africa (CAPRISA), University of KwaZulu–Natal, Durban, South Africa;; c eThekwini Municipality Health Unit, Durban, South Africa;; d KwaZulu-Natal Research and Innovation Sequencing Platform (KRISP), University of KwaZulu-Natal, Durban, South Africa;; e Department of Virology, University of KwaZulu-Natal and National Health Laboratory Service, Inkosi Albert Luthuli Central Hospital, KwaZulu-Natal, South Africa;; f Department of Global Health, Schools of Medicine and Public Health, University of Washington, Seattle, WA;; g Department of Medicine, School of Medicine, University of Washington, Seattle, WA;; h Department of Epidemiology, School of Public Health, University of Washington, Seattle, WA; and; i Discipline of Public Health Medicine, School of Nursing and Public Health, University of KwaZulu-Natal, Durban, South Africa.

**Keywords:** HIV, point-of-care, viral load, viral failure, primary care

## Abstract

Data are required regarding the feasibility of conducting a randomized trial of point-of-care viral load (VL) testing to guide management of HIV viremia and to provide estimates of effect to guide potential future trial design.

**Setting:**

Two public South African clinics during the dolutegravir-based antiretroviral therapy (ART) rollout.

**Methods:**

We randomized adults receiving first-line ART, with recent VL ≥1000 copies/mL, in a 1:1 ratio to receive point-of-care Xpert HIV-1 VL versus standard-of-care laboratory VL testing after 12 weeks. Feasibility outcomes included proportions of eligible patients enrolled and completing follow-up and VL process outcomes. Estimates of effect were assessed using the trial primary outcome of VL <50 copies/mL after 24 weeks.

**Results:**

From August 2020 to March 2022, we enrolled 80 eligible participants, an estimated 24% of those eligible. 47 of 80 (58.8%) were women, and the median age was 38.5 years (interquartile range [IQR], 33–45). 44 of 80 (55.0%) were receiving dolutegravir, and 36 of 80 (465.0%) were receiving efavirenz. After 12 weeks, point-of-care participants received VL results after median 3.1 hours (IQR 2.6–3.8), versus 7 days (IQR 6–8, *P* < 0.001) in standard of care. Twelve-week follow-up VL was ≥1000 copies/mL in 13 of 39 (33.3%) point-of-care participants and in 16 of 41 (39.0%) standard-of-care participants; 11 of 13 (84.6%) and 12 of 16 (75.0%) switched to second-line ART. After 24 weeks, 76 of 80 (95.0%) completed follow-up. 27 of 39 (69.2% [95% CI: 53.4 to 81.4]) point-of-care participants achieved VL <50 copies/mL versus 29 of 40 (72.5% [57.0 to 83.9]) standard-of-care participants. Point-of-care participants had median 3 (IQR, 3–4) clinical visits versus 4 (IQR, 4–5) in standard-of-care participants (*P* < 0.001).

**Conclusions:**

It was feasible to conduct a trial of point-of-care VL testing to manage viremia. Point-of-care VL lead to quicker results and fewer clinical visits, but estimates of 24-week VL suppression were similar between arms.

**Trial Registration:**

Pan African Clinical Trials Registry: PACTR202001785886049.

## INTRODUCTION

The World Health Organization^1^ (WHO) recommends annual viral load (VL) testing to monitor antiretroviral therapy (ART) for people living with HIV (PLHIV). Identifying people with viremia is important because they are at risk of immunocompromise, morbidity and mortality, onward HIV transmission, and the potential development and transmission of HIV drug resistance.^2^ Viremia may be caused by inconsistent adherence to effective ART, which can be managed with adherence counselling, and/or HIV drug resistance, which requires a change to second-line ART. Although the importance of achieving rapid viral resuppression is clear, several studies in low- and middle-income countries (LMICs) have documented long periods of sustained viremia because of poor clinical management, with associated increased morbidity and mortality.^2–9^

In these settings, HIV services are predominantly provided in primary care, where delays in receiving VL results from centralized laboratories^10^ can further impede management of viremia. New diagnostic strategies to improve the detection of viremia and achieve rapid viral resuppression are needed. The World Health Organization defines point-of-care VL testing as testing conducted in the clinic with results provided to clinicians for management on the same day.^11,12^ This may lead to faster VL resuppression, by allowing immediate enhanced adherence counselling and rapid switching to second-line ART. Furthermore, point-of-care testing may reduce the burden of clinical visits for review of test results. To date, 2 point-of-care assays have been approved as accurate by the WHO for use in LMICs^13,14^ and have been evaluated in our setting.^15,16^ Clinical trials of these assays among children,^17^ adolescents,^18^ and adults^19–21^ have demonstrated shorter turnaround times, but effects on clinical outcomes have been mixed.

None of these trials focused on point-of-care testing among people with viremia, who may benefit the most from quicker access to VL results. We plan a future efficacy trial of a diagnostic intervention to improve resuppression among people with viremia and require further data to aid design of this trial. First, the global rollout of dolutegravir, which has a higher genetic barrier to resistance,^22^ means we require more data on the proportion of people likely to achieve viral resuppression with this new drug. We also require estimates of the potential effect size of point-of-care VL on viral resuppression to guide power calculations for the planned trial. Second, poor adherence is often associated with social and psychological issues, such as financial problems, unstable employment, migrant labor, alcohol abuse, and stigma,^23^ meaning people with viremia may be more likely to be from marginalized and/or disadvantaged groups. Therefore, we do not know whether it is feasible to rapidly enroll people with viremia in clinical trials in our setting and to follow-up them to successfully assess outcomes. Third, we do not know whether routine health care staff will successfully use point-of-care VL results in clinical management.

We therefore aimed to conduct a feasibility study of a randomized trial of point-of-care VL testing to improve management viremia in the context of the dolutegravir-based ART rollout in South Africa.

## METHODS AND ANALYSIS

### Trial Design

We conducted the POwER study, an open-label, individually randomized, feasibility study of point-of-care HIV VL testing among people with HIV viremia while receiving first-line ART. The full protocol has been previously published.^24^

### Setting and Participants

This study was planned to start in April 2020 at the Prince Cyril Zulu Communicable Disease Centre (PCZ CDC), a large, public clinic next to the central Durban transport hub, with support from the adjacent Centre for the AIDS Programme of Research in South Africa (CAPRISA) eThekwini Clinical Research Site. Owing to COVID-19–related challenges with enrolment, we decided to expand to an additional clinic, which opened in January 2022. This was a rural, medium-sized, primary care clinic in the uMgungundlovu District, Mafakathini Clinic, with support from the adjacent CAPRISA Vulindlela Clinical Research Site. The clinic is located in a rural part of the uMgungudlovu District. Both sites provide HIV, tuberculosis, and other primary care services and are in the province of KwaZulu-Natal, which has an estimated HIV prevalence of 27% among adults aged 15–49 years.^25^

HIV treatment is provided free at the point of service, according to South African Department of Health guidelines,^26^ which recommend routine HIV VL testing at 6 months after ART initiation, and then annually, unless viremia is detected. Before December 2019, the standard first-line ART regimen was tenofovir disoproxil fumarate, emtricitabine, and efavirenz (TEE). From December 2019 onward, a new regimen was introduced, containing tenofovir disoproxil fumarate, lamivudine, and dolutegravir (TLD). During the trial, people already receiving TEE with VL <50 copies/mL were recommended to switch to TLD.

People were eligible for enrolment into POwER if they were living with HIV, aged 18 years or older, receiving first-line efavirenz- or dolutegravir-based ART, had their latest VL ≥1000 copies/mL within the past 6 weeks, and had not received enhanced adherence counselling for this episode of viremia. Pregnant women were not eligible as guidelines for VL monitoring and management of viremia differ in pregnancy.^26^

### Randomization

After providing informed consent, eligible participants were randomized by a study nurse, using a preprogrammed electronic case report form, in a 1:1 ratio to the point-of-care arm or the standard-of-care arm. A statistician generated the allocation sequence using random numbers with variable block sizes. Randomization was stratified by ART regimen at enrolment (efavirenz- or dolutegravir-based). All study staff except the statistician were blinded to the allocation sequence. Clinical staff and participants were told the participant's allocation at enrolment.

### Interventions

**FIGURE 1.**
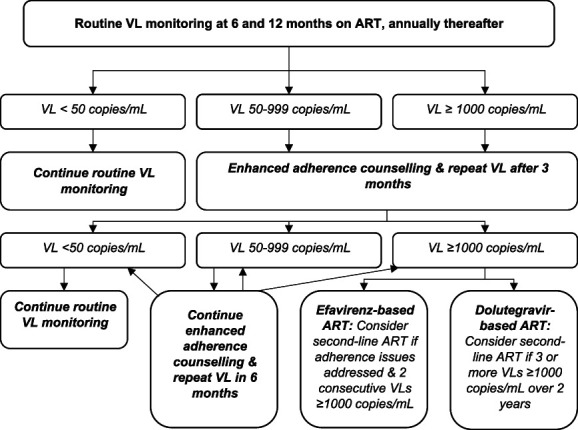
Management of viremia according to South African National Department of Health guidelines. Adapted from South African National Department of Health, 2019 ART Clinical Guidelines for the Management of HIV in Adults, Pregnancy, Adolescents, Children, Infants, and Neonates. Pretoria, South Africa, 2019.^26^

During follow-up until the exit visit 24 weeks after enrolment, clinical management was provided by public-sector clinical counsellors, HIV nurses, or clinicians according to the South African Department of Health guidelines, which specify that people with a VL >1000 copies/mL should receive enhanced adherence counselling and a repeat VL after 3 months (Fig. 1).^26^ The standard enhanced adherence counselling package is outlined in the South African guidelines and includes discussing VL results and treatment strategies and jointly identifying barriers and facilitators to adherence.^27^ After 3 months, if the repeat VL remains >1000 copies/mL, the guidelines recommend that people receiving efavirenz-based ART are switched to a second-line ART regimen because they are presumed to have HIV drug resistance (Fig. 1). If the repeat VL is <1000 copies/mL, then people receiving efavirenz-based regimens are recommended to transition to a dolutegravir-based first-line regimen. People receiving dolutegravir-based first-line regimens with a repeat VL of >1000 copies/mL are presumed to be more likely to experience viremia due to poor adherence because dolutegravir has a higher genetic barrier to resistance.^22^ They are therefore recommended to receive ongoing adherence support and only to consider switching to protease inhibitor–based second-line ART after 2 years of ongoing viremia, which is beyond the follow-up time of this study.

In both arms, stored plasma samples were taken at the enrolment, follow-up VL, and exit visits for retrospective VL and HIV drug resistance testing. Participants who were late for visits were tracked by clinical staff according to routine clinical procedures, which generally involve telephone follow-up if the participant is more than 14 days late.

#### Point-of-Care VL Testing

For participants randomized to the point-of-care VL intervention arm, all VLs during follow-up were conducted using the Xpert HIV-1 VL assay (Cepheid, Sunnyvale, CA). Initially, we planned for nurses to do the Xpert HIV-1 VL testing in the study clinic, but staff shortages and COVID-19–related disruption meant that it was conducted by a research laboratory technician in a clinical site laboratory in accordance with manufacturer instructions. In brief, a venous blood sample was centrifuged to provide 1 mL of plasma, which was tested using the Xpert HIV-1 VL cartridge. Participants were encouraged to wait for the result, which was provided to routine clinical staff to inform clinical management. If participants were not willing/able to wait, the results were provided at their next clinical appointment, scheduled by study staff in consultation with participants at the soonest possible date. In the case of invalid results, leftover plasma was used for retesting or a repeat sample was taken.

#### Laboratory-Based VL Testing

For participants randomized to the standard-of-care arm, all VL testing during follow-up was performed off-site by the National Health Laboratory Service, usually using the Alinity m HIV-1 VL analyzer (Abbott, Chicago, IL). Participants would receive the VL result at their next clinical appointment, typically scheduled as soon as possible after 1 week, to allow for laboratory VL turnaround time.

### Outcomes

To assess trial feasibility, we evaluated the proportion of potentially eligible participants who were enrolled, who completed follow-up, and who had same-day VL testing (point-of-care arm only). We obtained approvals to retrospectively assess routine, deidentified laboratory, and clinical data from PCZ CDC to determine the total number of potentially eligible participants during the study period, allowing us to calculate the proportion we managed to enroll. To provide exploratory estimates of the potential effect of point-of-care VL testing, we assessed the primary outcome of viral suppression <50 copies/mL at 24 weeks after enrolment in each arm according to intention-to-treat. Outcome VLs were measured retrospectively testing stored samples with the cobas HIV-1 assay on the cobas 6800 platform (Roche, Basel, Switzerland). Additional prespecified exploratory outcomes were HIV drug resistance in each arm at study exit, time to detection of viral failure (consecutive VLs ≥1000 copies/mL), switch to second-line ART, and appropriate switch/transition to dolutegravir-based ART.

### Sample Size and Statistical Methods

We estimated enrolling at least 100 participants in 6 months but allowed for flexibility to enroll 80–180 participants based on available time and resources. We calculated the proportions of participants achieving study outcomes and compared proportions in each arm using the Fisher exact test. For the primary outcome, participants who were lost-to-follow-up were included as not virally suppressed. We did not intend for the study to be powered to compare the primary outcome of viral suppression at 24 weeks between the 2 arms. For time-to-event outcomes, we assessed the median number of days from enrolment to each outcome and compared outcomes using Cox proportional hazards.

### Ethical Approvals

The University of KwaZulu-Natal Biomedical Research Ethics Committee (BREC 00000836/2019) and the University of Oxford Tropical Research Ethics Committee (OxTREC 66-19) approved this study. POwER is registered on the Pan African Clinical Trials Registry (PACTR202001785886049) and the South African Clinical Trials Registry (DOH-27-072020-6890).

## RESULTS

### Study Population

**FIGURE 2.**
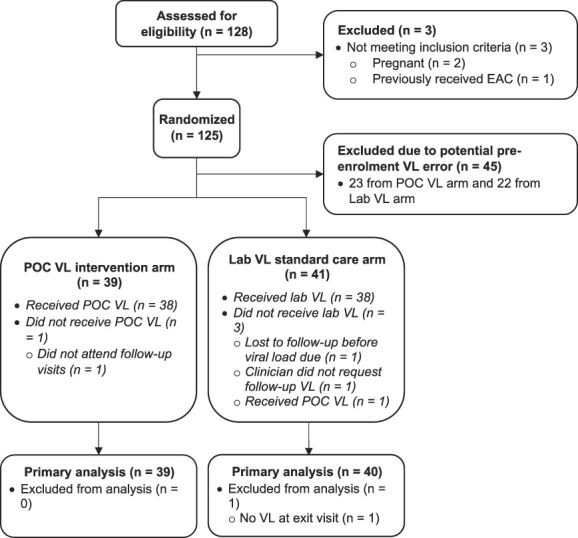
POWER study CONSORT diagram. EAC, enhanced adherence counselling; POC, point-of-care.

We enrolled the first participant on August 20, 2020. In December 2020, we were informed that before mid-October, 45 of 63 (71.4%) POwER participants had their routine pre-enrolment VLs measured on a defective Alinity m HIV-1 VL analyzer that overestimated some VL results. Therefore, the viremic sample used to determine eligibility may have been falsely high. After discussion with the ethics committees, we informed affected participants immediately and offered them to continue or terminate from this study; all opted to continue. We determined to exclude these participants from the primary analysis and to enroll a total of 125 participants (Fig. 2). None of the viral loads taken during follow-up were tested on the defective analyzer. There were no enrolments between December 2020–February 2021 and November 2021–January 2022 because of SARS-CoV-2 waves affecting South Africa. The last participant was enrolled on March 25, 2022, and completed the follow-up on September 7, 2022.

After excluding the stated 45 participants, we enrolled 80 participants who met the eligibility criteria. The median age was 38.5 years (interquartile range [IQR], 33–45), 58.8% were female, 78 of 80 (97.5%) were enroled at the PCZ CDC, and the median time on ART was 3.1 years (IQR, 1.0–5.7) (Table 1). At enrolment, 36 of 80 (45.0%) were receiving efavirenz and 44 of 80 (55.0%) were receiving dolutegravir-based ART. 17 of 44 (38.6%) had been initiated on dolutegravir, whereas the remaining 27 (61.4%) had been initiated on an efavirenz-based regimen and were subsequently transitioned to first-line dolutegravir. The median time on the dolutegravir-based regimen was 0.7 years (0.5–1.1). The median time since the viremic pre-enrolment VL was 15 days (range, 6–36), with 95% receiving enhanced adherence counselling at enrolment. On retrospective testing of enrolment samples, 45 participants remained viremic ≥1000 copies/mL.

**TABLE 1.** Baseline Characteristics of Eligible Participants Enrolled in the POwER Study, n = 80

Variable	Levels	Intervention Arm*	Standard-of-Care Arm*	Total*
Age, yrs	Median (IQR)	38.0 (33.0–45.5)	39.0 (33.0–43.0)	38.5 (33.0–45.0)
Sex	Female	20 (51.3)	27 (65.9)	47 (58.8)
	Male	19 (48.7)	14 (34.1)	33 (41.2)
Time since ART initiation, yrs	Median (IQR)	4.1 (1.0–5.1)	3.1 (1.0–7.0)	3.2 (1.0–6.0)
Current ART regimen at enrolment	TDF/3TC/DTG	22 (56.4)	21 (51.2)	43 (53.8)
	TDF/FTC/EFV	17 (43.6)	19 (46.3)	36 (45.0)
	ABC/3TC/DTG		1 (2.4)	1 (1.3)
Time on current regimen, yrs	Median (IQR)	1.1 (0.6–4.1)	1.0 (0.6–2.8)	1.1 (0.6–3.1)
Time on dolutegravir-based regimen, yrs	Median (IQR)	0.8 (0.5–1.2)	0.7 (0.5–1.0)	0.7 (0.5–1.1)
Comorbidities at enrolment?	Yes	5 (12.8)	4 (9.8)	9 (11.2)
ART side effects?	Yes	0 (0.0%)	2 (4.9)	2 (2.5)
Last time participant missed a dose of ART	Within past week	10 (25.6)	7 (17.1)	17 (21.2)
	1–2 wk ago	1 (2.6)	3 (7.3)	4 (5.0)
	2–4 wk ago	9 (23.1)	3 (7.3)	12 (15.0)
	1–3 mo ago	5 (12.8)	5 (12.2)	10 (12.5)
	>3 mo ago	0 (0.0)	2 (4.9)	2 (2.5)
	Never	14 (35.9)	21 (51.2)	35 (43.8)
Hazardous drinking (AUDIT-C)	Yes	12 (30.8)	14 (34.1)	26 (32.5)
Ethnicity	Black African	39 (100.0)	40 (97.6)	79 (98.8)
Highest level of education completed	Lower secondary	20 (51.3)	20 (48.8)	40 (50.0)
	Upper secondary (matriculation)	16 (41.0)	16 (39.0)	32 (40.0)
	Tertiary	3 (7.7)	5 (12.2)	8 (10.0)
Employment status	Unemployed	10 (25.6)	18 (43.9)	28 (35.0)
	Informal	4 (10.3)	2 (4.9)	6 (7.5)
	Part-time	5 (12.8)	4 (9.8)	9 (11.2)
	Full-time	16 (41.0)	11 (26.8)	27 (33.8)
	Student	3 (7.7)	2 (4.9)	5 (6.2)
	Self-employed	1 (2.6)	4 (9.8)	5 (6.2)
Monthly personal income, ZAR	<1000	16 (41.0)	20 (48.8)	36 (45.0)
	1000–4000	12 (30.8)	11 (26.8)	23 (28.8)
	4001–8000	8 (20.5)	8 (19.5)	16 (20.0)
	>8000	3 (7.7)	2 (4.9)	5 (6.2)
Travel time to clinic	≤30 min	18 (46.2)	24 (58.5)	42 (52.5)
	31–59 min	18 (46.2)	15 (36.6)	33 (41.2)
	1–2 h	3 (7.7)	2 (4.9)	5 (6.2)
Cost of travel to clinic and back (round trip), ZAR	0–25	13 (33.4)	17 (41.5)	30 (37.4)
	26–50	23 (59.0)	22 (53.7)	45 (56.2)
	> 50	3 (7.7)	2 (4.9)	5 (6.3)
Enrolment CD4 count, cells/µL	<200	10 (25.6)	11 (26.8)	21 (26.2)
	200–349	12 (30.8)	9 (22.0)	21 (26.2)
	350–499	8 (20.5)	10 (24.4)	18 (22.5)
	≥500	9 (23.1)	11 (26.8)	20 (25.0)
Time since viremic sample taken before enrolment, d	Median (IQR)	15.0 (14.0–20.5)	15.0 (13.0–21.0)	15.0 (13.0–21.0)
EAC at enrolment visit?	Yes	38 (97.4)	38 (92.7)	76 (95.0)
Enrolment viral load, copies/mL	<50	6 (15.4)	8 (19.5)	14 (17.5)
	50–999	11 (28.2)	10 (24.4)	21 (26.2)
	≥1000	22 (56.4)	23 (56.1)	45 (56.2)

* N, (%) unless stated otherwise.

EAC, enhanced adherence counselling.

In the standard-of-care arm versus the intervention arm, there were slightly higher proportions of women (27 of 41, 65.9% versus 20 of 39, 51.3%), people who reported never missing a dose of ART (21 of 41, 51.2% versus 14 of 39, 35.9%), unemployed people (18 of 41, 43.9% versus 10 of 39, 25.6%), and people with ≤30 minutes travel time to clinic (24 of 41, 58.5% versus 18 of 39, 46.2%). Other demographics and clinical variables were well-balanced between the 2 groups.

### Study Feasibility Outcomes

After excluding viral loads before mid-October 2020 which may have been affected by the defective analyzer, we retrospectively identified 262 nonpregnant adults with a true VL ≥1000 copies/mL who were receiving first-line ART between mid-October 2020 and March 2022 and may have been eligible for enrolment. During the same period, we were able to screen 65 and enroll 62 of 262 participants (23.7%), approximately 3.6 per month. The other 18 enrolments (to make the total of 80) occurred before mid-October 2020 and were known to not have been affected by the laboratory error. Overall, 76 of 80 participants (95.0%, 95% CI: 87.0 to 98.4) attended the study exit visit; 38 of 39 (97.4%, 95% CI: 84.9 to 99.9) in the intervention arm and 38 of 41 (92%, 95% CI: 79.0 to 98.1) in the standard-of-care arm. Two participants relocated, and 2 were lost to follow-up and not contactable.

### 24-Week Viral Suppression Outcome

After excluding one standard-of-care participant with no VL at their 24-week exit visit, 27 of 39, 69.2% participants (53.6–81.4) in the intervention arm and 29 of 40 72.5% (57.2–83.9) in the standard-of-care arm achieved the primary outcome of VL <50 copies/mL at 24 weeks (*P* = 0.808). Point estimates were similar when stratified by dolutegravir- and efavirenz-based regimens at enrolment (Table 2).

**TABLE 2.** POwER Study Outcomes, n = 80

Outcome	POC VL Intervention Arm n/N, % (95% CI)	Laboratory VL Standard-of-Care Arm n/N, % (95% CI)	*P*
Study feasibility outcomes			
Proportion of potentially eligible participants in PCZ CDC who were enrolled*	62/262, 23.7% (18.9 to –29.2)		
Proportion of enrolled participants who attended study exit visit	38/39, 97.4% (85.4 to -100)	38/41, 92.7%, (79.7 to -98.1)	0.616
VL process outcomes			
Median days from enrolment to follow-up VL	84 (IQR 84 to 96)	85 (IQR 78 to 91)	0.562
Time from follow-up VL blood draw to result with the patient	3.1 h (IQR 2.6 to 3.8)	7 d 0 h (IQR 6 d 18 h to 8 d 0 h)	<0.001
Proportion of VL results communicated to participant on the same day	33/38†, 86.8% (72.1 to 94.6)	1/39‡, 2.6% (0.0 to 14.6)	<0.001
Median number of visits during follow-up	3 (IQR 3 to 4)	4 (IQR 4 to 5)	<0.001
Mean number of EAC sessions during follow-up	1.51 (95% CI 1.29 to 1.73)	1.49 (95% CI 1.25 to 1.72)	
Follow-up VL (copies/mL)			
<50	22/39, 56.4% (41.0 to 70.7)	9/41, 22.0% (11.9 to 37.0)	0.003§
50–999	3/39, 7.7% (2.0 to 21.2)	14/41, 34.1% (21.6 to 49.5)	—
≥1000	13/39, 33.3% (20.6 to 49.1)	16/41, 39.0% (25.7 to 54.3)	—
Missing	1/39, 2.6% (0.0 to 14.6)	2/41, 4.9% (0.6 to 17.2%)	—
Viral failure outcomes			
Proportion of those with viral failure (repeat VL ≥1000 copies/mL) receiving same-day EAC	11/13, 84.6% (56.3 to 96.6)	0/16, 0.0% (0.0 to 23.1)	<0.001
Median days from enrolment to viral failure ≥1000 copies/mL result being given to the participant	85 (IQR 84 to 96)	95 (IQR 88.5 to 115)	0.038
Proportion of those with viral failure switched to second-line ART‖	11/13, 84.6% (56.3 to 96.6)	12/16, 75.0% (0.50 to 0.90)	0.663
Proportion of those with viral failure with same-day switch to second-line ART	8/11, 72.7% (42.8 to 90.5)	0/12, 0.0% (0.0 to 28.7)	<0.001
Median days from enrolment to switching to second-line ART	86 (IQR 84 to 92.5)	95 (IQR 89 to 103)	0.056
HIV drug resistance mutations detected at 24 wk	3/39, 7.7% (2.0 to 21.2)	3/41, 7.3% (1.9 to 20.3)	1.000
Estimate of effect on primary outcome of VL at 24 wk (copies/mL)			
24-week VL			
<50	27/39, 69.2% (53.4 to 81.4)	29/40¶, 72.5% (57.0 to 83.9)	0.808§
50-999	6/39, 15.4% (7.0 to 30.2)	5/40, 12.5% (5.1 to 26.7)	—
≥1000	5/39, 12.8% (5.2 to 27.3)	3/40, 7.5% (2.0 to 20.7)	—
Lost to follow-up	1/39, 2.6% (0.0 to 14.6)	3/40, 7.5% (2.0 to 20.7)	—
Receiving efavirenz at enrolment			
<50	12/17, 70.6% (46.5 to 86.8)	13/19, 68.4% (45.8 to 84.7)	—
50-999	3/17, 17.6% (5.6 to 42.0)	4/19, 21.1 (8.1 to 44.0)	—
≥1000	1/17, 5.9 (0.0 to 29.2)	1/19, 5.3 (0.0 to 26.8)	—
Lost to follow-up	1/17, 5.9 (0.0 to 29.2)	1/19, 5.3 (0.0 to 26.8)	—
Receiving dolutegravir at enrolment			
<50	15/22, 68.2% (47.1 to 83.7)	16/21, 76.2% (54.4 to 89.6)	—
50-999	3/22, 13.6 (4.1 to 34.4)	1/21, 4.8% (0.0 to 24.7)	—
≥1000	4/22, 18.2 (6.9 to 39.3)	2/21, 9.5% (1.6 to 30.4)	—
Lost to follow-up	0/22, 0.0% (0.0 to 17.9)	2/21, 9.5% (1.6 to 30.4)	—
Participants receiving efavirenz at enrolment who were changed to dolutegravir-based ART			
Primary analysis population (n=36)	16/17, 94.1% (70.7 to 100.0)	14/19, 73.7% (50.8 to 88.4)	0.183
All enrolled participants (n=74)	33/37, 89.2% (74.5 to 96.2)	24/37, 64.9%% (48.7 to 78.2)	0.025

* After excluding potentially falsely high VLs from before mid-October 2013, we retrospectively identified 262 participants who were potentially eligible for enrolment between October 13, 2020, and March 11, 2022. Sixty-two were enrolled in POwER, in addition to 18 who were enrolled before Oct 13th and confirmed to have true viremia, not affected by the falsely high viral load problem.

† Of the 5 not communicated on the same day, the results were communicated on days 1, 2, 2, 5, and 7, respectively, because of sample being drawn too late in the day for the result to be ready during opening hours (n = 1) and the participant not being able to wait for the result (n = 4).

‡ Viral load tested using point-of-care assay in error.

§ Comparing proportion with viral suppression <50 copies/mL in each arm.

‖ 11 of 13 (84.6%) with viral failure in the point-of-care arm, and 12 of 16 (68.8%) in the standard-of-care arm were receiving efavirenz-based first-line ART at the time of failure, all of those receiving efavirenz were switched to second-line ART.

¶ Excluding one participant who did not have a VL sample taken at the study exit visit.

### Viral Load Process Outcomes

**FIGURE 3.**
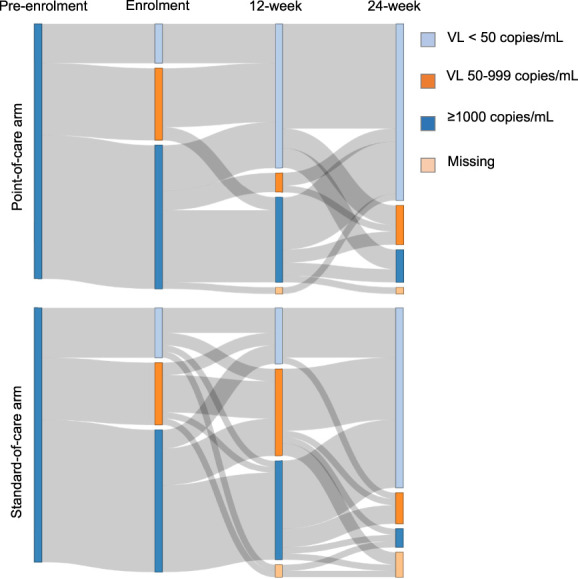
Changes in viral loads from pre-enrolment to study exit.

The proportion with 12-week follow-up VLs was high (intervention arm 97.4%, standard-of-care arm 95.1%), taken a median of 84 and 85 days from enrolment, respectively. 33 of 38 point-of-care participants (86.8%) received 12-week VL results on the same day, at a median time of 3.1 hours from blood draw, versus 7 days in the standard-of-care arm. One standard-of-care participant had their 12-week VL using the point-of-care assay in error. The proportion of patients with a 12-week VL <50 copies/mL was higher in the intervention (22 of 39, 56.4% [41.0–70.7]) versus the standard-of-care arm (9 of 41, 22.0% [12.0–36.7], *P* = 0.003), although proportions with viral failure (12-week VL >1000 copies/mL) were similar (Table 2, Fig. 3). Twelve-week viral failure was also much lower among participants receiving dolutegravir (7 of 44, 15.9%), compared with efavirenz (22 of 36, 61.1%). Among those with viral failure, the time to the participant receiving VL result was a median 10 days shorter in the intervention arm (Table 2). Of the 13 with viral failure in the intervention arm, 11 of 13, 84.6% (56.3–96.6) received same-day enhanced adherence counselling and 8 of 11, 72.7% (43.4–90.3) had same-day switch to second-line ART. The time from enrolment to second-line ART was 9 days shorter in the intervention arm (*P* = 0.056). Overall, there was a median of 3 follow-up visits (IQR 3–4) in the intervention arm versus 4 (IQR 4–5) in the standard-of-care arm (*P* < 0.001).

### Transition/Switch to Dolutegravir

Among the 36 participants in the primary analysis population who were on an efavirenz-based regimen at enrolment, 16 of 17 (94.1%, 95% CI: 70.7 to 100.0) in the intervention arm transitioned/switched to dolutegravir by 24 weeks versus 14 of 19, (73.7%, 95% CI: 50.8 to 88.4, *P* = 0.183) in the standard-of-care arm. Transition to first-line dolutegravir is particularly recommended for people with viral suppression, and therefore, we also assessed transition among all participants enrolled, including those who likely had a falsely high pre-enrolment VL. Transition was higher in the intervention arm (33 of 37, 89.2% [95% CI: 74.5 to 96.2] versus 24 of 37, 64.9% [95% CI: 48.7 to 78.2], *P* = 0.025).

## DISCUSSION

### Summary

In this feasibility study for a randomized trial of point-of-care VL testing to manage viremia, enrolment was challenging in the context of the COVID-19 pandemic, but once enrolled, follow-up was high. There was good fidelity to the point-of-care intervention, with faster time to results, quicker switching to second-line ART, and fewer clinical visits in the point-of-care arm. However, although not powered for a formal comparison, point estimates of viral suppression at 24 weeks were similar in the point-of-care and standard-of-care arms.

### Interpretation

The major feasibility challenge was enrolment, with an estimated 23.7% of potentially eligible patients enrolled. Disruptions during COVID-19 waves, with staff shortages due to illness/self-isolation, sometimes halted recruitment efforts and hampered clinical processes to track and call back patients with viremia for referral to study staff. It also led to delays that meant we were only able to open the second study site once we had nearly enrolled to target. Therefore, only 2 participants were enrolled at this site. Among participants who were enrolled, retention was good. It may be that these participants were more engaged in the clinic, and therefore more likely to be retained in care, than the average person with viremia.

This study was not powered to detect an improvement in the primary outcome of viral suppression between the point-of-care and standard laboratory testing arms, where we cannot rule out a difference. However, the point estimates were very similar between the 2 arms. Point-of-care testing was generally conducted in a timely manner, but there are several reasons which could explain why this did not translate into a difference in the point estimates of 24-week viral resuppression. First, viremia was managed well in the standard-of-care arm. The results were provided to participants after a median 7 days and those requiring second-line ART were switched after a median 10 days. These results contrast sharply with other South African studies that found long delays of 14–68 weeks until switching to second-line ART.^5,8,9,28^ Although we attempted to avoid influencing clinical management in this study, it is likely that being included in POwER increased focus on these patients, thereby improving the standard-of-care. We have previously observed this same phenomenon in an evaluation of a point-of-care versus laboratory testing tuberculosis diagnostic strategy.^29^ Second, the small sample size may have exaggerated imbalances in the baseline characteristics between the 2 arms, such as self-reported adherence, which may have favored the standard-of-care arm. Third, viral failure at 12 weeks (ie, at the time of the point-of-care intervention) was already low (36.3%), particularly among people receiving dolutegravir (16.3%), meaning there was less potential for the intervention to improve outcomes. A systematic review of people with viremia on first-line nonnucleoside reverse transcriptase inhibitors (such as efavirenz) found that just more than 50% developed viral failure,^30^ with the remainder resuppressing after adherence counselling alone. Our findings suggest that much lower viral failure on dolutegravir means there is less of a role for interventions that improve switching to second-line ART.

Among participants who were receiving efavirenz-based regimens at enrolment, a higher proportion were transitioned/switched to dolutegravir in the intervention arm. This was likely because of the higher proportion of participants with 12-week VL <50 copies/mL in this arm, making clinicians more comfortable to transition to dolutegravir. Reasons for the difference in 12-week VL suppression between the point-of-care and laboratory assay are not clear because we have found the Xpert HIV-1 VL to be sensitive and specific at a VL threshold of 50 copies/mL.^16^

### Comparison With Other Studies

Several trials of point-of-care VL testing have been published with mixed results. Our previous trial of point-of-care VL testing combined with task-shifting found an improvement of 13.9% in the combined outcome of 12-month viral suppression and retention in care, as well as increased availability and quicker turnaround time of VL results.^21^ In this study, all participants were on first-line efavirenz-based regimens, there were few participants with viral failure (16 of 390, 4.1%), and the research team conducted clinical management in the intervention arm, meaning it is difficult to determine the contribution of point-of-care testing within the overall implementation strategy. Trials among children in Kenya,^17^ adolescents in Haiti,^18^ and adults in Nigeria^19,20^ found that point-of-care VL testing improved time to receipt of VL results by 2, 4, and 20 weeks, respectively, with improved switching to second-line ART. However, none of these trials found a clear impact on viral suppression.

### Strengths and Limitations

Strengths of our study include the high fidelity to the point-of-care VL intervention and the use of routine health care staff and settings, with the management following South African and WHO guidelines. Our study was severely affected by the COVID-19 pandemic, which delayed study start and made it harder to consistently enroll participants. Although we were able to demonstrate the accuracy of nurse-led point-of-care testing in a substudy,^16^ we were not able to implement nurse-led testing in POwER as planned and conducted testing in a site laboratory instead. These difficulties, and some of our findings, may not be generalizable once the COVID-19 pandemic subsides. Despite these challenges, follow-up was good, as was delivery of point-of-care viral load results to participants. Although the dolutegravir rollout may have affected the potential for point-of-care VL testing to influence outcomes, we provide early data on viral resuppression among people with viremia who are receiving dolutegravir, which will help guide the development of interventions. Our study was conducted in South Africa, with most participants enrolled in an urban setting, meaning our findings may not be generalizable to other settings with less well-developed laboratory systems. Finally, we targeted people who had already been identified as viremic using a laboratory-based assay, meaning point-of-care VL was only used for the 12-week VL, with participants already having received enhanced adherence counselling at enrolment. Identifying people at risk of viremia using other methods, and then conducting the initial and 12-week VL using point-of-care assays, may provide more opportunities for point-of-care testing to influence outcomes.

### Implications for Research and Policy

Our findings suggest that a larger randomized trial of point-of-care VL testing to manage viremia may be feasible, but would likely need to be conducted at multiple sites to ensure adequate enrolment. Furthermore, our findings highlight the difficulty in evaluating diagnostic interventions that require changes in clinical flow, without inadvertently improving standard of care. Cluster-randomized trials may be more appropriate for evaluating this kind of intervention, although it is harder to generate small-scale preliminary data. In the context of the ongoing dolutegravir-based ART rollout, the opportunity for point-of-care testing to influence management of viremia^31^ (eg, switching to second-line ART) may also be reduced. Combining point-of-care VL with other interventions that improve the detection and management of adherence may also be a promising strategy.^32^ The reduced number of visits with point-of-care testing also suggests that further work should explore health care utilization and cost outcomes^33–35^ from the health care service and service user perspectives and also whether point-of-care viral load testing can improve differentiated ART delivery services. We will report findings from a qualitative assessment of point-of-care viral load testing in POwER in a separate manuscript.

## CONCLUSIONS

Apart from COVID-19–related challenges, it was feasible to conduct a trial of point-of-care VL testing to manage viremia. Point-of-care testing reduced time to results and clinical visits, but point-estimates of 24-week viral resuppression were similar to the standard-of-care arm. Larger studies are needed to assess clinical outcomes and to identify potential interventions for people with viremia in the context of the ongoing dolutegravir rollout.

## References

[1] World Health Organization. Consolidated Guidelines on HIV Prevention, Testing, Treatment, Service Delivery and Monitoring: Recommendations for a Public Health Approach. Geneva, Switzerland: World Health Organization; 2021. 34370423

[2] Murphy RA Court R Maartens G . Second-line antiretroviral therapy in sub-Saharan Africa: it is time to mind the gaps. AIDS Res Hum Retroviruses. 2017;33:1181–1184. 28793781 10.1089/aid.2017.0134 PMC5709698

[3] Teeraananchai S Law M Boettiger D . Virological failure and treatment switch after ART initiation among people living with HIV with and without routine viral load monitoring in Asia. J Int AIDS Soc. 2022;25:e25989. 36028921 10.1002/jia2.25989 PMC9418417

[4] Pham MD Nguyen HV Anderson D . Viral load monitoring for people living with HIV in the era of test and treat: progress made and challenges ahead—a systematic review. BMC Public Health. 2022;22:1203. 35710413 10.1186/s12889-022-13504-2 PMC9202111

[5] Hermans LE Carmona S Nijhuis M . Virological suppression and clinical management in response to Viremia in South African HIV treatment program: a multicenter cohort study. PLoS Med. 2020;17:e1003037. 32097428 10.1371/journal.pmed.1003037 PMC7041795

[6] Glass TR Motaboli L Nsakala B . The viral load monitoring cascade in a resource-limited setting: a prospective multicentre cohort study after introduction of routine viral load monitoring in rural Lesotho. PLoS One. 2019;14:e0220337. 31461455 10.1371/journal.pone.0220337 PMC6713472

[7] Iwuji C Shahmanesh M Koole O . Clinical outcomes after first-line HIV treatment failure in South Africa: the next cascade of care. HIV Med. 2020;21:457–462. 32495515 10.1111/hiv.12877 PMC7384088

[8] Bell Gorrod H Fox MP Boulle A . The impact of delayed switch to second-line antiretroviral therapy on mortality, depending on failure time definition and CD4 count at failure. Am J Epidemiol. 2020;189:811–819. 32219384 10.1093/aje/kwaa049 PMC7523585

[9] Bell Gorrod H Court R Schomaker M . Increased mortality with delayed and missed switch to second-line antiretroviral therapy in South Africa. J Acquir Immune Defic Syndr. 2020;84:107–113. 32032304 10.1097/QAI.0000000000002313 PMC7269121

[10] Lubega P Nalugya SJ Kimuli AN . Adherence to viral load testing guidelines, barriers, and associated factors among persons living with HIV on ART in Southwestern Uganda: a mixed-methods study. BMC Public Health. 2022;22:1268. 35768800 10.1186/s12889-022-13674-z PMC9244194

[11] Drain PK Dorward J Bender A . Point-of-care HIV viral load testing: an essential tool for a sustainable global HIV/AIDS response. Clin Microbiol Rev. 2019;32:e00097–18. 31092508 10.1128/CMR.00097-18 PMC6589862

[12] Dorward J Drain PK Garrett N. Point-of-care viral load testing and differentiated HIV care. Lancet HIV. 2018;5:e8–e9. 29290227 10.1016/S2352-3018(17)30211-4 PMC6003416

[13] World Health Organization. WHO Prequalification of in Vitro Diagnostics. Product: m-PIMA HIV-1/2 VL. Geneva: World Health Organization; 2019.

[14] World Health Organization. WHO Prequalification of In Vitro Diagnostics. Product: Xpert® HIV-1 Viral Load with GeneXpert® Dx, GeneXpert® Infinity- 48, GeneXpert® Infinity-48s and GeneXpert® Infinity-80. Geneva: World Health Organization; 2017.

[15] Garrett NJ Drain PK Werner L . Diagnostic accuracy of the point-of-care Xpert HIV-1 viral load assay in a South African HIV clinic. J Acquir Immune Defic Syndr. 2016;72:e45–e48. 26959192 10.1097/QAI.0000000000000978 PMC4866899

[16] Dorward J Naidoo J Moodley P . Diagnostic accuracy of the rapid Xpert HIV-1 viral load XC, Xpert HIV-1 viral load, & m-PIMA HIV-1/2 viral load in South African clinics. J Acquir Immune Defic Syndr. 2022;91:189–196. 36094486 10.1097/QAI.0000000000003037 PMC7613592

[17] Patel RC Oyaro P Thomas KK . Point-of-care HIV viral load and targeted drug resistance mutation testing versus standard care for Kenyan children on antiretroviral therapy (Opt4Kids): an open-label, randomised controlled trial. Lancet Child Adolesc Health. 2022;6:681–691. 35987208 10.1016/S2352-4642(22)00191-2 PMC9482947

[18] Reif LK Belizaire ME Rouzier V . Point-of-care viral load testing among adolescents and young adults living with HIV in Haiti: a randomized control trial. AIDS Care. 2022;34:409–420. 34612092 10.1080/09540121.2021.1981816 PMC8976702

[19] Chaplin B Agbaji O Reyes Nieva H . Timeliness of point-of-care viral load results improves human immunodeficiency virus monitoring in Nigeria. Clin Infect Dis. 2022;76:e671–e680. 10.1093/cid/ciac609 PMC11150521 35872644

[20] Chang C Agbaji O Mitruka K . Clinical outcomes in a randomized controlled trial comparing point-of-care with standard human immunodeficiency virus (HIV) viral load monitoring in Nigeria. Clin Infect Dis. 2022;76:e681–e691. 10.1093/cid/ciac605 PMC11150517 35867672

[21] Drain PK Dorward J Violette LR . Point-of-care HIV viral load testing combined with task shifting to improve treatment outcomes (STREAM): findings from an open-label, non-inferiority, randomised controlled trial. Lancet HIV. 2020;7:e229–e237. 32105625 10.1016/S2352-3018(19)30402-3 PMC7183312

[22] Dorward J Lessells R Drain PK . Dolutegravir for first-line antiretroviral therapy in low-income and middle-income countries: uncertainties and opportunities for implementation and research. Lancet HIV. 2018;5:e400–e404. 29884404 10.1016/S2352-3018(18)30093-6 PMC6063784

[23] Shubber Z Mills EJ Nachega JB . Patient-reported barriers to adherence to antiretroviral therapy: a systematic review and meta-analysis. PLoS Med. 2016;13:e1002183. 27898679 10.1371/journal.pmed.1002183 PMC5127502

[24] Dorward J Sookrajh Y Ngobese H . Protocol for a randomised feasibility study of point-of-care HIV viral load testing to enhance re-suppression in South Africa: the POwER study. BMJ Open. 2021;11:e045373. 10.1136/bmjopen-2020-045373 PMC7888322 33593788

[25] Human Sciences Research Council. The Fifth South African National HIV Prevalence, Incidence, Behaviour and Communication Survey, 2017: HIV Impact Assessment Summary Report. Cape Town, South Africa: Human Sciences Research Council; 2018.

[26] The South African National Department of Health. 2019 ART Clinical Guidelines for the Management of HIV in Adults, Pregnancy, Adolescents, Children, Infants and Neonates. Pretoria, South Africa: The South African National Department of Health; 2019.

[27] The South African National Department of Health. Adherence Guidelines for HIV, TB and NCDs. Pretoria, South Africa, 2020. Available at: https://test.knowledgehub.org.za/elibrary/adherence-guidelines-hiv-tb-and-ncds-standard-operating-procedures-2020. Accessed May 11, 2023.

[28] Rohr JK Ive P Robert Horsburgh C . Marginal structural models to assess delays in second-line HIV treatment initiation in South Africa. PLoS One. 2016;11:1–11. 10.1371/journal.pone.0161469 PMC4993510 27548695

[29] Lessells RJ Cooke GS McGrath N . Impact of point-of-care Xpert MTB/RIF on tuberculosis treatment initiation. A cluster-randomized trial. Am J Respir Crit Care Med. 2017;196:901–910. 28727491 10.1164/rccm.201702-0278OC PMC5649979

[30] Ford N Orrell C Shubber Z . HIV viral resuppression following an elevated viral load: a systematic review and meta‐analysis. J Int AIDS Soc. 2019;22:e25415–e25416. 31746541 10.1002/jia2.25415 PMC6864498

[31] Ferrante di Ruffano L Hyde CJ McCaffery KJ . Assessing the value of diagnostic tests: a framework for designing and evaluating trials. BMJ. 2012;344:e686. 22354600 10.1136/bmj.e686

[32] Bardon AR Dorward J Sookrajh Y . Simplifying TREAtment and Monitoring for HIV (STREAM HIV): protocol for a randomised controlled trial of point-of-care urine tenofovir and viral load testing to improve HIV outcomes. BMJ Open. 2021;11:e050116. 10.1136/bmjopen-2021-050116 PMC8493905 34610939

[33] Simeon K Sharma M Dorward J . Comparative cost analysis of point-of-care versus laboratory-based testing to initiate and monitor HIV treatment in South Africa. PLoS One. 2019;14:e0223669. 31618220 10.1371/journal.pone.0223669 PMC6795460

[34] Sharma M Mudimu E Simeon K . Cost-effectiveness of point-of-care testing with task-shifting for HIV care in South Africa: a modelling study. Lancet HIV. 2021;8:e216–e224. 33347810 10.1016/S2352-3018(20)30279-4 PMC8284441

[35] Girdwood SJ Crompton T Sharma M . Cost-effectiveness of adoption strategies for point of care HIV viral load monitoring in South Africa. EClinicalMedicine. 2020;28:100607. 33294817 10.1016/j.eclinm.2020.100607 PMC7700965

